# Clinically relevant doses of vitamin A decrease cortical bone mass in mice

**DOI:** 10.1530/JOE-18-0316

**Published:** 2018-09-24

**Authors:** Vikte Lionikaite, Karin L Gustafsson, Anna Westerlund, Sara H Windahl, Antti Koskela, Juha Tuukkanen, Helena Johansson, Claes Ohlsson, H Herschel Conaway, Petra Henning, Ulf H Lerner

**Affiliations:** 1 Centre for Bone and Arthritis ResearchDepartment of Internal Medicine and Clinical Nutrition, Institute for Medicine, Sahlgrenska Academy at University of Gothenburg, Gothenburg, Sweden; 2 Department of Anatomy and Cell BiologyMedical Research Center, University of Oulu, Oulu, Finland; 3 Institute for Health and AgingCatholic University of Australia, Melbourne, Australia; 4 Department of Physiology and BiophysicsUniversity of Arkansas for Medical Sciences, Little Rock, Arkansas, USA

**Keywords:** vitamin A, osteoporosis, cortical bone, osteoclasts

## Abstract

Excess vitamin A has been associated with decreased cortical bone thickness and increased fracture risk. While most studies in rodents have employed high dosages of vitamin A for short periods of time, we investigated the bone phenotype in mice after longer exposure to more clinically relevant doses. For 1, 4 and 10 weeks, mice were fed a control diet (4.5 µg retinyl acetate/g chow), a diet modeled from the human upper tolerable limit (UTL; 20 µg retinyl acetate/g chow) and a diet three times UTL (supplemented; 60 µg retinyl acetate/g chow). Time-dependent decreases in periosteal circumference and bone mineral content were noted with the supplemented dose. These reductions in cortical bone resulted in a significant time-dependent decrease of predicted strength and a non-significant trend toward reduced bone strength as analyzed by three-point bending. Trabecular bone in tibiae and vertebrae remained unaffected when vitamin A was increased in the diet. Dynamic histomorphometry demonstrated that bone formation was substantially decreased after 1 week of treatment at the periosteal site with the supplemental dose. Increasing amount of vitamin A decreased endocortical circumference, resulting in decreased marrow area, a response associated with enhanced endocortical bone formation. In the presence of bisphosphonate, vitamin A had no effect on cortical bone, suggesting that osteoclasts are important, even if effects on bone resorption were not detected by osteoclast counting, genes in cortical bone or analysis of serum TRAP5b and CTX. In conclusion, our results indicate that even clinically relevant doses of vitamin A have a negative impact on the amount of cortical bone.

## Introduction

Vitamin A is an essential nutrient consumed in the diet in the form of retinyl esters or beta carotene. Retinyl esters are transported by chylomicrons to the liver where they are converted to retinol and bound to retinol-binding protein and released into the blood stream. In target cells, retinol is converted to all-trans retinoic acid (ATRA), which is the hormonally active form of vitamin A. ATRA binds to cellular retinoic acid-binding protein and translocates to the nucleus where it ligates primarily to retinoic acid receptors, regulating gene transcription (reviewed in Tata 2002, Conaway *et al.* 2013, Henning *et al.* 2015, Green *et al.* 2016). ATRA can also diffuse to adjacent cells and have paracrine effects and acts as an important morphogen during embryonic development (Kam *et al.* 2012). Vitamin A plays a crucial role in various physiological functions, including immune system regulation, vision and cell growth and differentiation (reviewed in Wiseman *et al.* 2017).

In humans, increased vitamin A consumption and elevated serum retinol levels have been associated with increased bone fragility and fracture risk (Melhus *et al.* 1998, Whiting & Lemke 1999, Feskanich *et al.* 2002, Michaelsson *et al.* 2003), suggesting that increased intake of vitamin A may be a risk factor for secondary osteoporosis. Although not all studies have consistently demonstrated the negative relationship between vitamin A and bone mass (for review see Conaway *et al.* 2013), a meta-analysis has shown that increased intake of vitamin A and elevated blood levels of retinol are associated with an increased risk of hip fracture (Wu *et al.* 2014). Interestingly, a dose–response analysis revealed that decreased levels of retinol also increased the risk of hip fracture (Wu *et al.* 2014).

Rodent studies have also shown detrimental effects of vitamin A on the skeleton. Rapid induction of hypervitaminosis A, either by feeding or by injecting retinoids, induces cortical bone loss in long bones of rats (Trechsel *et al.* 1987, Hough *et al.* 1988, Johansson *et al.* 2002, Kneissel *et al.* 2005, Lind *et al.* 2011, 2013, Wray *et al.* 2011) and mice (Kneissel *et al.* 2005), which is associated with decreased bone strength (Johansson *et al.* 2002, Lind *et al.* 2011). This reduction in the amount of cortical bone has been attributed to an increase in the number of periosteal osteoclasts (Trechsel *et al.* 1987, Hough *et al.* 1988, Kneissel *et al.* 2005, Lind *et al.* 2011). In trabecular bone, there have been fewer studies investigating the effects of vitamin A and the results have been inconsistent. In two studies, trabecular bone mineral density in the femur and the tibia was decreased by increased vitamin A intake (Lind *et al.* 2011, Wray *et al.* 2011), whereas in three other studies no effects on the trabecular bone mineral density of the humerus or tibia were observed (Johansson *et al.* 2002, Kneissel *et al.* 2005, Green *et al.* 2015). In the only study investigating the effect of increased dietary retinol on trabecular bone mass in vertebrae, it was found that vitamin A decreased bone mass, an effect associated with both an increased number of osteoclasts and a decreased number of osteoblasts (Yorgan *et al.* 2016).

Although numerous investigations have shown either stimulatory or inhibitory effects of vitamin A on osteoblast differentiation and function *in vitro* (reviewed in Conaway *et al.* 2013, Green *et al.* 2016), very few studies have analyzed effects of vitamin A on bone formation *in vivo*. Kneissel *et al.* and Lind* et al.* found that hypervitaminosis A decreased mineralizing surfaces on the periosteal side of cortical bone in the femur and tibia (Kneissel *et al.* 2005, Lind *et al.* 2013); however, mineralizing apposition rate was inhibited in one of these studies (Lind *et al.* 2013), but unaffected in the other (Kneissel *et al.* 2005).

The current recommended daily allowance (RDA) of vitamin A in adults is 900 and 700 µg retinol activity equivalents per day in males and females, respectively, while the UTL is 3000 µg/day (Trumbo *et al.* 2001). With over one-third of the population in the USA taking dietary supplements (Bailey *et al.* 2011), excess vitamin A consumption/hypervitaminosis A is a potential health risk.

Although rodent studies have provided insight into changes in cortical bone caused by hypervitaminosis A, the doses (13 to at least 142 times higher than the human RDA diet) and the short durations (7–14 days) of the experiments are questionable reflections of human consumption. For this reason, the present study was designed to investigate the effects of lower, more clinically relevant dosages of vitamin A (4.5–13 times higher modeled from the human RDA) on bone phenotype in mice after longer exposure (1–10 weeks). We assessed in detail not only the effects of vitamin A on the amount of cortical bone, but also vitamin A’s effects on trabecular bone in long bones as well as in vertebra, paying special attention to osteoclastic bone resorption and osteoblastic bone formation.

## Materials and methods

### Animals and study design

All animal experimental procedures were approved by the Ethics Committee at the University of Gothenburg and carried out in accordance to relevant guidelines. C57BL/6 female mice (Harlan Laboratories, Inc.; Taconic Bioscience) were received at 7–8 weeks of age and acclimatized for 1 week. C57BL6/J mice of 9–19 weeks, as used in this experiment, are sexually mature at the start of the experiments and growing less rapidly than mice aged 5–8 weeks. Mice were housed in groups of five at 22°C with a 12:12 h light–darkness cycle and fed diets (Harlan Laboratories Inc.) containing either 4.5 µg retinyl acetate/g chow (control (Harlan 2016) repelleted), 20 µg retinyl acetate/g chow (upper tolerable level; UTL) or 60 µg retinyl acetate/g chow (supplemented) *ad libitum* for 4 (*n* = 15/group) or 10 weeks (*n* = 10/group). By using a 4.5- and 13-fold increased concentrations of retinyl acetate in the chow, we aimed to mimic the fold increase for upper tolerable and supplemented levels in humans calculated from the RDA. An additional 8-day experiment with three groups was performed, feeding 8- to 9-week-old mice control, supplemented or hypervitaminosis A diet (Hypervit A; 450 µg retinyl acetate/g chow, *n* = 10/group).

A separate bisphosphonate experiment was performed on 8- to 9-week-old mice pre-treated with intraperitoneal (i.p.) injections of 200 µL zoledronic acid (ZA; 200 µg/kg; 0.02 µg/µL; Frasenius Kabi AB, Uppsala, Sweden) or vehicle (saline) twice/week for 2 weeks (*n* = 10/group) (de Molon *et al.* 2015). They were then fed control (4.5 µg retinyl acetate/g chow) or supplemented (60 µg retinyl acetate/g chow) vitamin A diets and the twice/week injections of ZA continued for 4 weeks.

Body weights were monitored throughout all experiments (Supplementary Fig. 1, see section on supplementary data given at the end of this article). At the termination of all experiments, mice were anesthetized with Ketador/Dexdomitor cocktail. Blood was collected from the subclavian artery. Serum was aliquoted and stored at −80°C till further use. Mice were terminated via cervical dislocation. Liver was dissected and weighed. Vertebra (L3–L6), femurs and tibias were dissected, fixed in formalin for 3 days and stored in ethanol for later analysis.

The predesigned primary endpoints were to record the effect of vitamin A diets on cortical and trabecular bone mass. All assessments of the outcomes were done in total blinding of the investigators. Power analysis suggested that we needed at least eight mice per group to detect biologically significant effects.

### Serum analyses

Serum retinol and retinyl ester (RE) analysis was carried out by Vital Analytical Services (Oslo, Norway) using high-performance liquid chromatography. Serum tartrate-resistant acid phosphatase (TRAP), C-terminal type I collagen (CTX) and osteocalcin (OCN) were measured using the MouseTRAP (TRAcP 5b) ELISA kit (Immunodiagnostic System), RatLaps (CTX-I) EIA ELISA kit (Immunodiagnostic System) and Osteocalcin ELISA kit (Immutopics, Inc.), respectively, following manufacturer’s instructions.

### Quantitative PCR

The vertebral body of the L3 and L6 (trabecular bone) and the flushed mid-diaphyseal tibias (cortical bone) were stored at −80°C in RNAlater post killing. RNA was extracted using TRIzol reagent (Life Technologies) followed by the RNeasy mini kit (Qiagen). Single-strand cDNA was synthesized using a High-Capacity cDNA Reverse Transcription kit (Applied Biosystems). Quantitative real-time PCR (qPCR) analyses were performed by using predesigned Taqman Assays and Taqman Fast Advance Master Mix (Applied Biosystems). The following predesigned real-time PCR assays were used for gene expression analysis: *Acp5* (*Trap*; Mm00475698_m1), *Ctsk* (Mm00484036_m1), *Tnfsf11* (*Rankl*; Mm00441908_m1), *Alpl* (*Alp*; Mm00475834_m1), *Tnfrs11a* (*Rank*; Mm00437135_m1), *Tnfrsf11b* (*Opg*; Mm0043545_m1), *Bglap* (*Osteocalcin*; Mm01741771_g1), *Col1α1* (Mm00801666_g1), *Runx2* (Mm00501580_m1), *Sp7* (*Osterix*; Mm04209856_m1) (Applied Biosystems). The house-keeping gene 18S was used as the endogenous control in all analyses. Data are displayed as fold change relative to control.

### Assessment of bone parameters

#### Peripheral quantitative computed tomography (pQCT) and microcomputed tomography (µCT)

Peripheral quantitative computed tomography (pQCT) was run on the femur and tibia with the pQCT XCT RESEARCH M (version 4.5B; Norland). The voxel size was 70 µm isotropically. The growth plate was located by a scout scan, and the boundary of the metaphysis and growth plate was used as a reference point for trabecular and cortical scans. Trabecular bone mineral density (Tb.BMD) was determined with a single metaphyseal scan of the proximal tibia, at a distance 2.6% of the total tibia bone length distal from the growth plate, or of the distal femur, corresponding to a distance 3% of the total femur bone length proximal from the growth plate. Trabecular bone region was defined as the inner 45% of the total cross-sectional area to avoid contamination with cortical bone. Cortical bone parameters were analyzed by a single scan in the approximate mid-diaphyseal region of the tibia, at a distance 30% of the total tibia bone length distal from the proximal growth plate or of the femur, at a distance 36% of the total femur bone length proximal from the distal growth plate. Cortical bone mineral content is presented as mg/mm bone length calculated from the diaphyseal scan. Cortical thickness was determined by the software (Stratec XCT, Research M pQCT v. 6.20C) as the average thickness using a circular model with the threshold set to 710 mg/cm^3^. Bone length was measured using a caliper. 

Microcomputed tomography (µCT) analysis was performed on the vertebra (L5) using the Skyscan 1072 scanner (Bruker MicroCT, Aartselaar, Belgium) and imaged with an X-ray tube voltage of 50 kV and current of 201 µA with an 0.5 mm aluminum filter. Transverse scanning angular rotation was 180° and the angular increment of 0.70°. The voxel size was 13.5 µm isotropically. The NRecon software (Burker) was used for image reconstruction following established guidelines (Bouxsein *et al.* 2010).

#### Histomorphometry and measurement of mechanical strength

For the measure of dynamic bone parameters, mice were injected (i.p.) with 100 µL of calcein (50 mg/kg) 8 and 1 day before termination. The whole femurs were fixed in 4% paraformaldehyde, followed by dehydration in 70% ethanol and embedded in methyl meta-acrylate. Twenty-five micrometer transverse sections of cortical bone from the approximate mid-diaphyseal region were cut and periosteal and endocortical mineralized surface/bone surface (MS/BS), mineral apposition rate (MAR) and bone formation rate/bone surface (BFR/BS) were analyzed.

For static cellular bone parameters, femurs were fixed in 4% phosphate-buffered paraformaldehyde, decalcified in 10% EDTA in Tris-buffer pH 6.95 and embedded in paraffin. Coronal sections were stained with heamatotoxylin and TRAP-positive osteoclasts were detected by the Naphol AS-BI method. Trabecular bone osteoclasts were assessed at the distal and proximal femur in the metaphyseal area, not including osteoclasts near the growth plate. Cortical bone osteoclasts were counted in the diaphysis, excluding the metaphysis.

Histological measurements of marrow area (Ma.Ar), total area (Tt.Ar) and cortical bone area (Ct.Ar) were acquired from sections used for dynamic histomorphometry.

All parameters (dynamic and static) were measured using the Osteomeasure Histomorphometry System (OsteoMetrics) following the guidelines of the American Society for Bone and Mineral Research (Dempster *et al.* 2013).

Mechanical strength was assessed in the tibias from 10-week control and supplemented vitamin A mice using the three-point bending test. Supports were placed ±2.75–2.8 mm from 50% of the total length of the bone, totaling the span length of 5.5 mm. Tests were performed at a loading speed of 0.155 mm/s with a mechanical testing machine (Instron 3366, Instron) at mid-tibia, with the press head loaded on the medial side. Results were calculated with custom-made Excel macros based on the computer recorded load deformation raw data curves before the first point of permanent deformation, produced by Bluehill 2 software v2.6 (Insitron). From the raw data curves, the *F*(max) is the maximum loading force (N) applied till breaking, and stiffness (N/mm) was the slope of the load deformation curve.

### Statistical analyses

Figures and tables are presented as mean ± standard error of the mean (s.e.m.). D’Agostino-Pearson omnibus normality test was used to assess normality of data and equal variance was assumed. Student’s *t*-test and one-way ANOVA with Dunnett’s *post hoc* test were used to analyze treatment effect. Two-way ANOVA for interaction was used to study the time-dependent effect of vitamin A treatment and the result of ZA treatment. No adjustment was made for multiple comparison testing other than Dunnett’s. 95% CIs of the effect are displayed where appropriate. GraphPad Prism 7 statistical software v7.02 (GraphPad Software, Inc) was used with *P* < 0.05 considered statistically significant. A multivariable linear regression model was fitted to 4- and 10-week treatment data using both dosage (4.5, 20 and 60 μg retinyl acetate/g chow) and duration (4 and 10 weeks) of treatment as independent variables, and bone mineral content (BMC), cortical thickness and periosteal and endocortical circumference as dependent variable in each model. The interaction between dosage and duration was used as an additional independent variable. In a linear regression model, the association between dosage (4.5, 20 and 60 µg/g) and cortical bone mass is linear and the model cannot detect a non-linear association, i.e. the reported decrease of cortical bone mass per 10 µg/g increase is estimated from the overall association between cortical bone mass and the dosage in the three treatment groups.

## Results

### Vitamin A-treated mice appear healthy with increased relative liver weights and increased serum retinol and retinyl ester levels

After 4 and 10 weeks of treatment with UTL or supplemented doses of vitamin A, mice appeared healthy with no significant difference in body weight change between treatment groups and control (Table 1 and Supplementary Fig. 1). Liver weight, as percentage of body weight, was increased at 4 and 10 weeks in the supplemented dose group compared to control mice (Table 1). Bone length of the tibia and femur was not significantly affected by the UTL or supplemental doses compared to controls (Table 1).

**Table 1:** Final body weight, relative liver weight, femur and tibia length.

	4 weeks (*n* = 15/group)	10 weeks (*n* = 10/group)
Final body weight (g)		
Control	18.2 ± 0.3	20.9 ± 0.5
UTL	18.6 ± 0.3	20.2 ± 0.5
Supplemented	18.2 ± 0.3	20.7 ± 0.5
Liver weight (%)		
Control	4.3 ± 0.09	4.2 ± 0.06
UTL	4.6 ± 0.07	4.5 ± 0.07
Supplemented	4.8 ± 0.13**	4.6 ± 0.12**
Tibia length (mm)		
Control	16.8 ± 0.1	17.3 ± 0.15
UTL	16.9 ± 0.08	17.3 ± 0.19
Supplemented	16.9 ± 0.13	17.6± 0.17
Femur length (mm)		
Control	14.4 ± 0.11	14.8 ± 0.17
UTL	14.6 ± 0.09	14.9 ± 0.15
Supplemented	14.5 ± 0.1	15.0 ± 0.12

Final body weight, liver weight (% of body weight) and tibia and femur length after 4 and 10 weeks of UTL and supplemented diets. Values given as mean ± s.e.m.

**P < 0.01: 1-way ANOVA followed by Dunnett’s multiple comparison test vs respective control.

**Figure 1:**
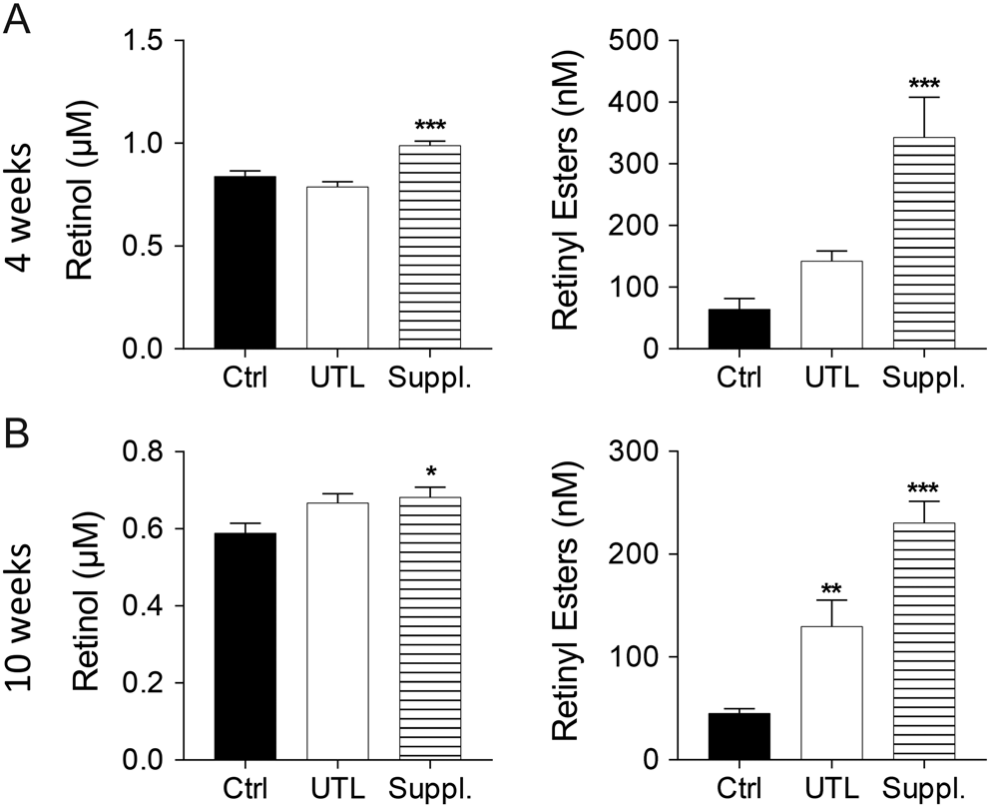
Vitamin A supplementation increases serum retinol and retinyl ester (RE) levels. Serum retinol and RE at 4 (A) and 10 (B) weeks. Values given as mean ± s.e.m. (*n* = 10/group). 1-way ANOVA followed by Dunnett’s multiple comparison test vs control, *P < 0.05, **P < 0.01, ***P < 0.001.

Mice fed control chow containing 4.5 µg retinyl acetate/g chow for 4 weeks had serum levels of 0.84 ± 0.03 µM retinol and 65 ± 17 nM retinyl esters, which is similar to what has been previously observed in C57BL/6 mice (Obrochta *et al.* 2014). Serum retinol was increased significantly by the supplemented dose of vitamin A in comparison to control at both time points, with the UTL diet showing no significant change (Fig. 1). The serum RE (the sum of retinyl linoleate, retinyl palmitate, retinyl oleate, retinyl stearate) levels were significantly higher than the RE in mice fed control chow after both 4 and 10 weeks of supplemental vitamin A diet. Mice fed UTL chow displayed significantly increased serum RE levels at 10 weeks (Fig. 1).

### Vitamin A decreases cortical bone parameters in a time-dependent manner

**Figure 2:**
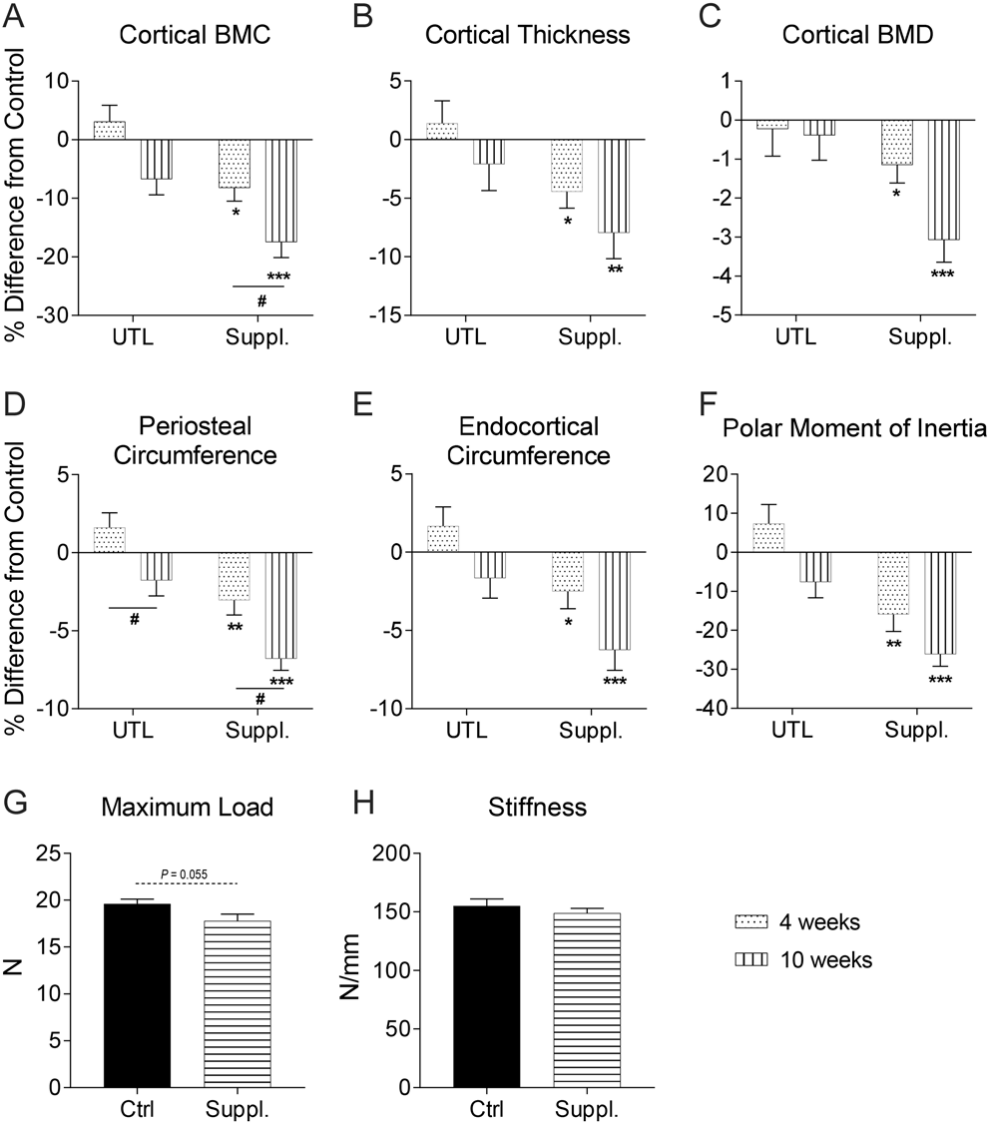
Supplemented vitamin A dose, but not UTL dose, affects cortical bone parameters of the tibia in a time-dependent manner and decreases predicted bone strength. UTL and supplemented diet effect on (A) cortical bone mineral content (BMC), (B) cortical thickness, (C) cortical bone mineral density (BMD), (D) periosteal circumference, (E) endocortical circumference, and (F) polar moment of inertia of the tibia. pQCT results in % difference vs respective control. Three-point bending of the tibia after 10 weeks of supplemented diet (G) maximum load applied till failure (*P* = 0.055) and (H) stiffness. (A, B, C, D, E and F) Student’s *t*-test vs respective controls, *P < 0.05, **P < 0.01, ***P < 0.001. 2-way ANOVA for interaction, ^#^*P* < 0.05, ^##^*P* < 0.01. *n* = 15/group at 4 weeks, *n* = 10/group at 10 weeks. (G and H) Student’s *t*-test, *n* = 10/group. All values displayed as mean ± s.e.m.

After 4 weeks of supplemental vitamin A diet, cortical BMC, cortical thickness and cortical bone mineral density (BMD) were lower in the tibia (Fig. 2A, B and C) as assessed by pQCT. After 10 weeks of treatment, the effects on cortical BMC were significantly more pronounced (Fig. 2A). Both periosteal and endocortical circumferences were significantly decreased after 4 weeks with the supplemented diet (Fig. 2D and E). The decrease in periosteal circumference progressively increased after 10 weeks (Fig. 2D). Similar results were also obtained in the femur (Supplementary Fig. 2A, B, C, D, E and F). The UTL diet caused a significant decrease in periosteal circumference in both the tibia (Fig. 2D) and the femur (Supplementary Fig. 2F) over time.

These changes in cortical bone led to a significant decrease in mean polar moment of inertia (predicted strength) assessed by pQCT, in both the tibia (Fig. 2F) and the femur (Supplementary Fig. 2F) after 4 and 10 weeks of treatment with the supplemented diet, but not the UTL diet. Formal strength testing (three-point bending) was performed on tibias from 10-week supplemental diet, since the phenotype observed was more pronounced than at 4 weeks. Supplemented vitamin A diet resulted in a trend of lower bone strength (−9%; *P* = 0.055; Fig. 2G), while the stiffness remained unchanged between the two groups (Fig. 2H).

Linear regression analyses, using data from the three treatment groups (4.5, 20 and 60 µg retinyl acetate/g chow) and both treatment time points (4 and 10 weeks), showed that femur cortical BMC and thickness, as well as periosteal and endocortical circumference are significantly associated with the dosage of vitamin A when the data are adjusted for treatment time (Table 2). There was no statistical significant interaction between dosage and time for any cortical bone parameter (*P* > 0.14).

**Table 2:** Linear regression analysis indicates that increases of vitamin A in the chow is significantly associated with lower cortical bone parameters when adjusted for number of weeks of treatment.

	Change per 10 µg/g increase in dose (95% CI)
Cortical BMC (mg/mm)	−0.016 (−0.023, −0.0085)***
Cortical thickness (µm)	−0.0012 (−0.0023, −0.0001)*
Periosteal circumference (mm)	−0.040 (−0.053, −0.026)***
Endocortical circumference (mm)	−0.032 (−0.047, −0.017)***

Values given as change per 10 µg/g increase in dose with 95% confidence intervals (95% CI).

*P < 0.05, ***P < 0.001.

### Supplemental vitamin A diet does not affect trabecular bone

Trabecular bone mass in tibia was not significantly changed after 4 and 10 weeks of UTL and supplemented vitamin A chow compared to control, as assessed by pQCT (Table 3). To further analyze trabecular bone, microCT analysis was performed on the L5 vertebra. Results demonstrated that trabecular bone volume fraction in the vertebrae was not significantly affected with the supplemented vitamin A diet compared to control in the present study (Table 3).

**Table 3:** UTL and supplemental vitamin A diets did not affect trabecular bone.

	Tibia	Vertebra			
	Tb.BMD	BV/TV	Tb.N	Tb.Th	Tb.Sp
4 weeks	(*n* = 15/group)	(*n* = 10/group)			
Control	209 ± 6	22.8 ± 1.1	4.96 ± 0.2	46 ± 1	93 ± 3
UTL	211 ± 6 (−20.7, 24.6)	–	–	–	–
Supplemented	219 ± 9 (−12.1, 33.2)	23.3 ± 1.2 (−2.91, 3.75)	5.22 ± 0.2 (−0.33, 0.85)	45 ± 1 (−4.02, 0.96)	96 ± 4 (−7.94, 13.4)
10 weeks	(*n* = 10/group)	(*n* = 10/group)			
Control	200 ± 8	22.1 ± 1.0	4.77 ± 0.2	47 ± 0.7	158 ± 4
UTL	210 ± 12 (−26.3, 45.0)	–	–	–	–
Supplemented	179 ± 12 (−56.5, 14.8)	22.4 ± 1.4 (−3.37, 3.79)	5.02 ± 0.3 (−0.53, 1.04)	45 ± 1 (−4.54, 0.43)	155 ± 5 (−16.2, 10.2)

Trabecular bone mineral density (Tb. BMD; mg/cm^3^), as analyzed by pQCT, in tibia at 4 and 10 weeks of UTL and supplemented vitamin A diet. Vertebral bone volume/tissue volume (BV/TV; %), trabecular number (Tb.N; /mm), thickness (Tb.Th; µm) and separation (Tb.Sp; µm) of the L5 vertebra via µCT after 4 and 10 weeks of supplemented vitamin A treatment and the 95% CI of the effect in brackets. Values are given as mean ± s.e.m. 1-way ANOVA followed by Dunnett’s multiple comparison test vs control for tibia pQCT and Student’s *t*-test vs control for µCT of vertebra.

### Expression analysis of osteoclastic and osteoblastic genes in cortical bone

The mRNA expression of osteoclastic genes in cortical bone of the tibia (*Acp5*, *Ctsk*, *Tnfrsf11a*, *Tnfsf11*, *Tnfrsf11b*) was not affected after 4 or 10 weeks of supplemented diet (Table 4). At 4 weeks, the mRNA expression of the osteoblastic genes osteocalcin (*Bglap*) and alkaline phosphatase (*Alpl*) were decreased after both supplemented (Table 4) and UTL (data not shown) dosages of vitamin A, whereas other osteoblastic genes, such as *Col1a1*, *Runx2*, and *Sp7*, were not significantly regulated.

**Table 4:** Expression analysis of osteoclastic and osteoblastic genes in cortical bone after 4 and 10 weeks of supplemented vitamin A diet.

	4 week		CI of effect	10 week		CI of effect
	Control	Supplemented		Control	Supplemented	
*Acp5 (Trap)*	1.00 ± 0.16	0.78 ± 0.07	−0.58, 0.14	1.00 ± 0.10	0.89 ± 0.08	−0.38, 0.17
*Ctsk*	1.00 ± 0.17	0.81 ± 0.08	−0.59, 0.21	1.00 ± 0.11	0.83 ± 0.06	−0.44, 0.09
*Tnfrsf11a (Rank)*	1.00 ± 0.12	0.97 ± 0.09	−0.35, 0.29	1.00 ± 0.10	0.90 ± 0.05	−0.34, 0.14
*Tnfsf11 (Rankl)*	1.00 ± 0.17	0.90 ± 0.07	−0.49, 0.29	1.00 ± 0.13	1.03 ± 0.13	−0.35, 0.42
*Tnfrsf11b (Opg)*	1.00 ± 0.20	0.79 ± 0.12	−0.69, 0.27	1.00 ± 0.06	1.39 ± 0.19	−0.03, 0.82
*Bglap (Ocn)*	1.00 ± 0.18	0.49 ± 0.08*	−0.93, −0.09	1.00 ± 0.10	1.05 ± 0.18	−0.37, 0.48
*Alpl*	1.00 ± 0.15	0.63 ± 0.06*	−0.70, −0.04	1.00 ± 0.07	1.13 ± 0.17	−0.25, 0.51
*Col1α1*	1.00 ± 0.17	0.82 ± 0.07	−0.57, 0.21	1.00 ± 0.10	1.10 ± 0.17	−0.31, 0.52
*Runx2*	1.00 ± 0.20	0.60 ± 0.07	−0.84, 0.04	1.00 ± 0.07	1.06 ± 0.06	−0.14, 0.25
*Sp7 (Osterix)*	1.00 ± 0.13	1.35 ± 0.20	−0.14, 0.85	1.00 ± 0.06	1.18 ± 0.11	−0.09, 0.45

Osteoclastic (*Acp5*, *Ctsk*, *Tnfrsf11a*, *Tnfsf11*, and *Tnfrsf11b*) and osteoblastic (*Bglap*, *Alpl*, *Col1α1*, *Runx2*, and *Sp7*) genes at 4 and 10 weeks of supplemented vitamin A treatment and the 95% CI of the effect. Data is displayed as fold change relative to control, *n* = 10/group, mean ± s.e.m., Student’s *t*-test vs control.

*P < 0.05.

In vertebra, the mRNA expression of *Acp5*, *Ctsk*, *Tnfrsf11a*, *Tnfsf11*, *Tnfrsf11b*, *Bglap*, *Alpl*, *Col1a*, *Runx2* and *Sp7* were not significantly regulated by the supplemented diet group after 10 weeks (data not shown).

### Serum resorption and formation markers indicate no effect on osteoclast or osteoblast activity

Analysis of serum TRAP5b and CTX indicated no significant differences in osteoclast activity at 4 or 10 weeks of supplemented vitamin A diet (Supplementary Table 1). Osteoblast activity also appeared to be unchanged when analyzed by serum levels of osteocalcin (Supplementary Table 1).

### Histomorphometric analysis indicates no significant change in the number of osteoclasts

TRAP stained longitudinal sections of the femurs of mice exposed to supplemented diet for 10 weeks revealed no significant change in the number of osteoclasts present on either the periosteal, endocortical, or trabecular bone (Supplementary Table 2).

### Dynamic histomorphometry shows that long-term vitamin A transiently increases endocortical bone formation rate with no effect on the periosteal surface

Dynamic histomorphometry measurements showed that endocortical MAR and bone formation rate (BFR/BS) were increased in the femur after 4 weeks of supplemented diet, but no significant effect on mineralization surfaces (MS/BS) was noted in the present study (Table 5). After 10 weeks of the diet, these increases were no longer observed; however, marrow area was decreased by 31% compared to controls (Table 5). In contrast, MS/BS, MAR or BFR/BS at the periosteal surface were not significantly affected at 4 and 10 weeks, although the total area was decreased by 19% after 10 weeks (Table 5).

**Table 5:** Dynamic histology shows vitamin A transiently increases endocortical bone formation after 4 weeks of supplemented vitamin A diet. Histological analysis indicates smaller marrow area and total area after 10 weeks of supplemental diet.

	4 weeks		10 weeks		Interaction
	Control	Supplemented	Control	Supplemented	
Periosteal					
MS/BS (%)	30.5 ± 2.27	22.0 ± 3.44	31.2 ± 3.85	29.2 ± 1.81	n.s.
MAR (µm/day)	0.72 ± 0.06	0.59 ± 0.07	0.67 ± 0.04	0.64 ± 0.07	n.s.
BFR/BS (µm^3^/µm^2^/day)	0.22 ± 0.03	0.19 ± 0.06	0.23 ± 0.04	0.19 ± 0.02	n.s.
Endocortical					
MS/BS (%)	76.4 ± 1.40	81.2 ± 2.83	74.0 ± 3.20	81.4 ± 3.38	n.s.
MAR (µm/day)	2.18 ± 0.20	2.82 ± 0.10*	1.38 ± 0.05	1.39 ± 0.11	*P* < 0.05
BFR/BS (µm^3^/µm^2^/day)	1.66 ± 0.15	2.28 ± 0.08**	1.02 ± 0.05	1.16 ± 0.11	*P* < 0.05
Ma.Ar (mm^2^)	0.52 ± 0.03	0.55 ± 0.03	0.74 ± 0.03	0.51 ± 0.03***	*P* < 0.001
Tt.Ar (mm^2^)	0.96 ± 0.04	0.96 ± 0.04	1.48 ± 0.05	1.2 ± 0.03***	*P* < 0.01
Ct.Ar (mm^2^)	0.44 ± 0.01	0.41 ± 0.01	0.74 ± 0.03	0.69 ± 0.01	n.s.

Femur periosteal and endocortical mineralized surface/bone surface (MS/BS), mineral apposition rate (MAR), and bone formation rate/bone surface (BFR/BS) obtained from dynamic histomorphometry, and marrow area (Ma.Ar), total area (Tt.Ar), and cortical bone area (Ct.Ar) after 4 and 10 weeks of supplemented vitamin A diet. Values represented as mean ± s.e.m., *n* = 10/group, Student’s *t*-test vs control, 2-way ANOVA for interactions, n.s. indicates no statistical significance.

*P < 0.05, **P < 0.01, ***P < 0.001.

### Short duration supplemented and hypervitaminosis A diet decrease cortical bone parameters

We observed that the periosteal and endocortical circumferences, as well as cortical BMC, were reduced after 8 days in mice fed supplemented vitamin A diet when assessed by pQCT (Table 6).

**Table 6:** Short duration supplemented vitamin A diet decreased endocortical circumference and hypervitaminosis A diet decreased cortical bone parameters.

	Control	Supplemented	Hypervitaminosis A
Final body weight (g)	18.6 ± 0.3	18.1 ± 0.2	17.3 ± 0.2**
Liver weight (%)	4.9 ± 0.1	4.9 ± 0.09	5.3 ± 0.1**
Femoral length (mm)	14.2 ± 0.07	14.2 ± 0.05	14.3 ± 0.07
Tb. BMD (mg/cm^3^)	387.8 ± 9.83	347.3 ± 17.3	360.0 ± 15.5
Endocortical circumference (mm)	3.28 ± 0.03	3.16 ± 0.02**	3.07 ± 0.03***
Periosteal circumference (mm)	4.94 ± 0.03	4.81 ± 0.03**	4.51 ± 0.03***
Cortical BMC (mg/mm)	0.86 ± 0.01	0.83 ± 0.01*	0.64 ± 0.02***
Cortical thickness (µm)	264 ± 3	262 ± 2	229 ± 5***

Final body and relative liver weight, femoral length, trabecular bone mineral density (Tb. BMD), endocortical and periosteal circumferences, cortical bone mineral content (BMC), and cortical thickness as assessed by pQCT of the femur after 8 days of supplemented and hypervitaminosis A diet. Values represented as mean ± s.e.m., *n* = 10/group, 1-way ANOVA followed by Dunnett’s multiple comparison test vs control.

*P < 0.05, **P < 0.01, ***P < 0.001.

Hypervitaminosis A treatment (450 µg retinyl acetate/g chow) induced a much larger effect on bone phenotype than those observed with supplemented diet, illustrated by more pronounced decreases in several cortical bone parameters in the femur, including cortical BMC and cortical thickness, as well as periosteal and endocortical circumferences, without effects on longitudinal bone growth (Table 6). We could not detect any significant effect on the trabecular BMD (95% CI of effect: −78.8, 23.2) in the metaphyseal area of the femur, as assessed by pQCT, by hypervitaminosis A diet (Table 6). Similar cortical and trabecular parameters were also observed in the tibia (data not shown). Mice treated with the hypervitaminosis A diet had decreased final body weight and increased relative liver weight compared to control (Table 6).

### Short duration hypervitaminosis A increases the number of periosteal osteoclasts and decreases endocortical osteoclasts

**Figure 3:**
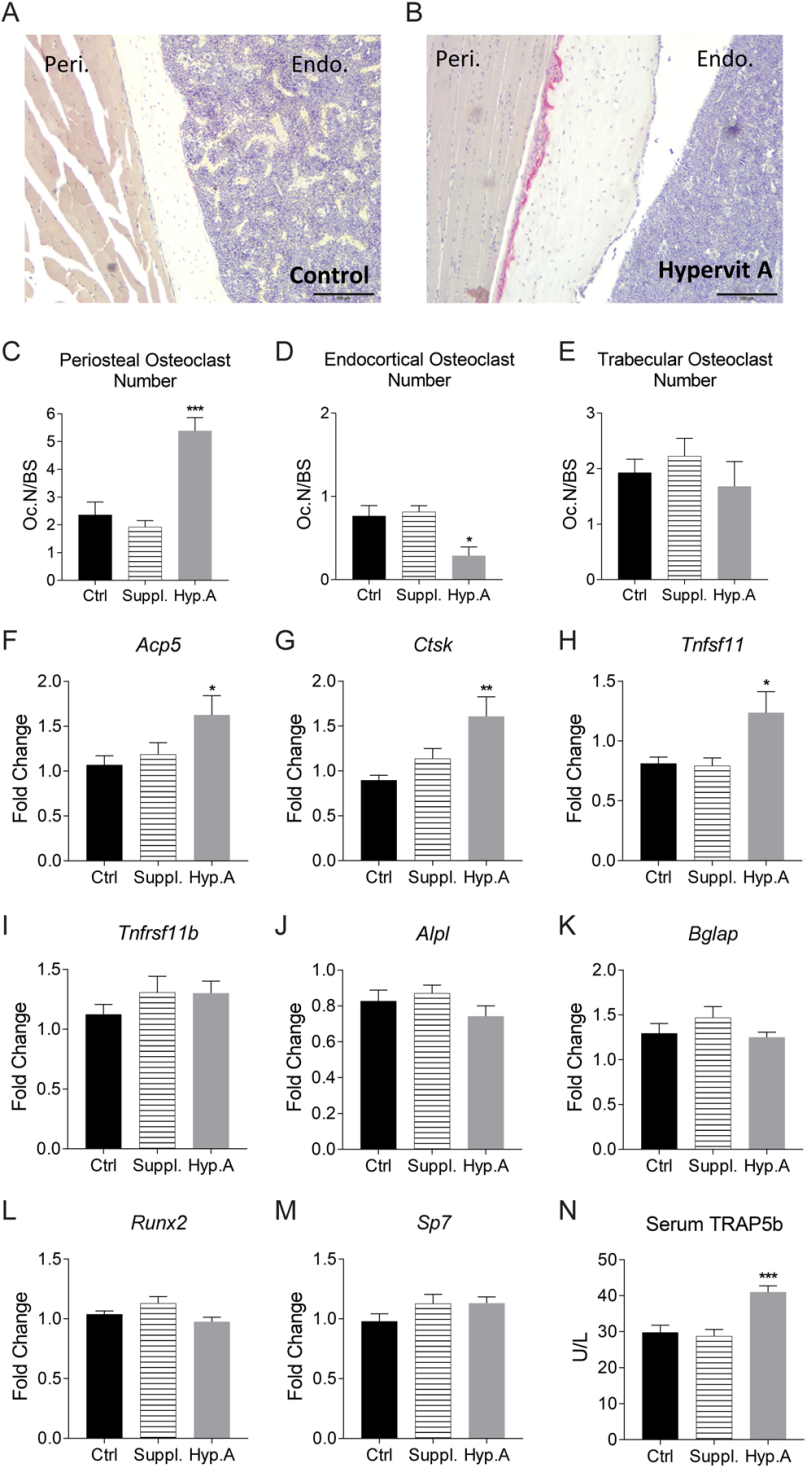
Short duration hypervitaminosis A experiment decreased cortical bone parameters, an effect caused by an increase in osteoclast number. Representative images of TRAP stained sections of femur of (A) control and (B) hypervitaminosis A treated mice for 8 days, scale bars represent 100 µm. Osteoclast numbers per measured bone surface counted from the (C) periosteum, (D) endosteum, and (E) trabeculae in TRAP stained femur sections of control (*n* = 10), supplemental (*n* = 9), and hypervitaminosis A (*n* = 5) mice after 8 days. Gene expression from cortical bone of the tibia of osteoclastic genes (F) *Acp5* (encodes for Trap), (G) *Ctsk*, (H) *Tnfsf11* (encodes for Rankl), (I) *Tnfrsf11b* (encodes for Opg) and osteoblastic genes (J) *Alpl*, (K) *Bglap* (encodes for Osteocalcin), (L) *Runx2*, and (M) *Sp7* (encodes for Osterix) in fold change relative to control. Serum analysis of (N) TRAP5b. Figures displayed as mean ± s.e.m., 1-way ANOVA followed by Dunnett’s multiple comparison test vs control, *P < 0.05, **P < 0.01, ***P < 0.001, *n* = 10/group.

Static histomorphometry of femurs in mice fed a hypervitaminosis A diet showed increases in the number of osteoclasts on periosteal bone and decreases on endocortical bone, but no effect on trabecular osteoclast numbers (Fig. 3A, B, C, D and E). In contrast, no significant changes in osteoclast numbers were observed in mice fed the supplemented vitamin A diet (Fig. 3C, D and E).

### Short duration hypervitaminosis A increases osteoclastic gene expression and serum Trap5b

Cortical bone gene expression in tibia of the osteoclast genes *Acp5*, *Ctsk* and *Tnfsf11* increased with hypervitaminosis A treatment compared to control, but was not significantly changed by supplemental vitamin A diet compared to control (Fig. 3F, G and H). This stimulatory effect by hypervitaminosis A was mimicked by serum TRAP5b analysis (Fig. 3N).

### Short-term supplemented dose of vitamin A affects periosteal and endocortical bone formation

Osteoblast genes (*Tnfrsf11b*, *Alpl*, *Bglap*, *Runx2* and* Sp7*) remained unchanged with both the supplemented dose and hypervitaminosis A treatment (Fig. 3I, J, K, L and M). Importantly, dynamic histomorphometry in mice fed the supplemented vitamin A diet for 8 days showed a pronounced decrease in periosteal BFR (−61%, *P*** **< 0.05; Table 7). In contrast, endocortical BFR was increased by the supplemented diet (Table 7).

**Table 7:** Dynamic histology shows vitamin A decreased periosteal bone formation and increased endocortical bone formation after 8 days of supplemented vitamin A diet.

	Control (*n* = 10)	Supplemented (*n* = 8)
Periosteal		
MS/BS (%)	23.2 ± 4.34	18.1 ± 3.98
MAR (µm/day)	2.16 ± 0.55	0.91 ± 0.11*P* ^ = 0.062^
BFR/BS (µm^3^/µm^2^/day)	0.47 ± 0.10	0.18 ± 0.06*
Endocortical		
MS/BS (%)	85.7 ± 2.4	88.3 ± 1.8
MAR (µm/day)	5.38 ± 0.26	6.50 ± 0.31*
BFR/BS (µm^3^/µm^2^/day)	4.63 ± 0.30	5.76 ± 0.36*

Femur periosteal and endocortical mineralized surface/bone surface (MS/BS), mineral apposition rate (MAR), and bone formation rate/bone surface (BFR/BS) after 8 days of supplemented vitamin A diet obtained from dynamic histomorphometry. Values represented as mean ± s.e.m. Student’s *t*-test.

*P < 0.05.

### Vitamin A effects on cortical bone were not observed in the presence of bisphosphonate treatment

**Figure 4:**
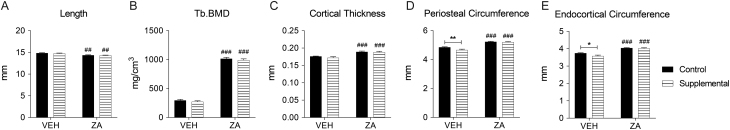
Vitamin A effects on cortical bone were not observed in the presence of bisphosphonate treatment. Femur (A) length, (B) trabecular bone mineral density (Tb. BMD), (C) cortical bone thickness, and (D) periosteal and (E) endocortical circumference as measured by pQCT. Similar results observed in tibia. Values given as mean ± s.e.m., *n* = 10/group. Student’s *t*-test; Vehicle (VEH): control vs supplemented, and Zoledronic acid (ZA): control vs supplemented *P < 0.05, **P < 0.01. Student’s *t*-test ZA treatment vs respective VEH control, ^#^*P* < 0.05, ^##^*P* < 0.01, ^###^*P* < 0.001. 2-way ANOVA for interaction, *P* > 0.05.

No significant effects of vitamin A on periosteal or endocortical circumferences was observed when ZA-treated mice were fed supplemented vitamin A chow (Fig. 4D and E). Treatment with ZA resulted in decreased bone length (Fig. 4A) and increased trabecular BMD and cortical thickness (Fig. 4B and C) compared to vehicle controls. Mice treated with ZA appeared healthy throughout the experiment and the treatment did not affect body weight change (Supplementary Fig. 1C).

## Discussion

The main findings of this study are as follows: (1) Experiments of 10-week duration with diets containing either 20 or 60 µg retinyl acetate/g of chow resulted in dose-dependent increases of circulating levels of RE equal to 130 and 231 nM, respectively. These are levels below or close to levels suggested for hypervitaminosis A in humans (200 nM) (Krasinski *et al.* 1989). (2) Increases in vitamin A levels time-dependently decreased cortical bone mass resulting in a trend toward decreased strength, (3) supplemental vitamin A diet did not significantly affect trabecular bone phenotype in the tibia or vertebra, (4) increased osteoclastic bone resorption, in combination with an early decrease in periosteal bone formation resulted in decreased periosteal circumference and (5) enhanced endocortical bone formation resulted in decreased marrow area and endocortical circumference.

The predominant form of vitamin A in the serum is retinol with normal physiological levels of 2–4 µM in humans and 1 µM in mice (Krasinski *et al.* 1989, Michaelsson *et al.* 2003, O’Byrne & Blaner 2013, Obrochta *et al.* 2014). In the present study, mice fed the supplemental diet had increased serum retinol levels. This method of determining vitamin A status is frequently used in human assessment; however, retinol levels in the serum are not reflective of vitamin A status unless there is a deficiency or surplus of the nutrient. Serum retinyl esters have been shown to be a more precise measurement of vitamin A status (Croquet *et al.* 2000). The normal physiological levels of retinyl esters in humans are in the range of 50–200 nM (Krasinski *et al.* 1989, Ballew *et al.* 2001), and it has been suggested that RE levels over 200 nM or exceeding 10% of total serum vitamin A (retinol and RE) may indicate excess vitamin A stores and potential vitamin A toxicity (Krasinski *et al.* 1989), although a specific cutoff has been difficult to determine (Ballew *et al.* 2001). In our experiments, 4 and 10 weeks of supplemented vitamin A diet resulted in RE levels of 343 ± 65 nM and 231 ± 21 nM, respectively, which exceeds the suggested RE threshold set for humans and indicates potential vitamin A excess.

The UTL dose used in the present study (20 µg retinyl acetate/g chow) resulted in RE levels of 130–143 nM at 4 and 10 weeks. This dose, modeled after the human UTL, by definition should be the maximum vitamin A consumption that does not cause ill effects. Although no statistically significant bone phenotype compared to control was observed, there was a trend for effects on all cortical bone parameters observed after 10, but not after 4 weeks, which is reflected by a significant decrease of periosteal circumference in the femur and tibia at 10 weeks when compared to the circumference at 4 weeks. The effects at 10 weeks were also similar, though not as pronounced as those noted with the supplemented diet (60 µg retinyl acetate/g chow) at 10 weeks.

Linear regression analysis showed that there was a significant association of increased vitamin A dosage (4.5, 20 and 60 µg retinyl acetate/g chow) and decreased cortical bone parameters. These observations indicate that even small increases of the vitamin A dose may negatively affect cortical bone parameters although the effects of the UTL dose, when tested separately, did not reach statistical significance.

Supplemented vitamin A diet caused a decrease in periosteal and endocortical circumferences of bone after 8 days, and at 4 and 10 weeks, with the severity of the periosteal phenotype increasing with time. Cortical BMC also mimicked this effect. These findings are consistent with previous studies, regardless of doses, administration or animal model used (Johansson *et al.* 2002, Kneissel *et al.* 2005, Lind *et al.* 2006, 2011, 2013). Cortical thickness significantly decreased after 10 weeks of supplemented vitamin A diet in the present study, and collectively, the results of our study indicate smaller bones.

In human studies, associations have been made between elevated vitamin A intake and decreased bone strength (Melhus *et al.* 1998, Feskanich *et al.* 2002, Promislow *et al.* 2002, Michaelsson *et al.* 2003); however, it is important to note that not all studies have observed this and on the contrary, inverse (Barker *et al.* 2005, Maggio *et al.* 2006) or no (Sowers & Wallace 1990, Wolf *et al.* 2005) association between increased vitamin A intake and fracture risk have also been reported. A meta-analysis of 12 prospective studies has recently concluded that both high and low levels of serum retinol, but not intake of β-carotene, are associated with increased risk of hip fracture, but not the risk of all fractures in the skeleton (Wu *et al.* 2014). These findings are in agreement with our observations that increased vitamin A decreases cortical but not trabecular bone. Johansson *et al.* investigated ‘subclinical hypervitaminosis A’ doses in rats and observed that 180 µg retinyl acetate and palmitate/g pellet for 12 weeks resulted in a significant reduction of breaking force compared to controls (Johansson *et al.* 2002). A short-duration (7-day) hypervitaminosis A (510 µg retinyl acetate and palmitate/g pellet) study in rats done by Lind *et al.* also observed a reduction in bone strength (Lind *et al.* 2011). These data, along with our findings, indicate weaker bones with increased vitamin A intake, even at clinically relevant doses although a certain threshold of vitamin A status most likely must be reached. Our supplemented vitamin A dose (60 µg retinyl acetate/g chow) resulted in reduced bone strength calculated indirectly as polar moment of inertia from pQCT data. Three-point bending also indicated a trend in reduced bone strength (−9%) after 10 weeks; however, it was not statistically significant.

While a cortical phenotype is evident, both with our clinically relevant doses of vitamin A-treated mice, and previous rat hypervitaminosis A studies, fewer studies have addressed the trabecular bone phenotype. Kneissel* et al.* injected rats with 150 µg/kg of retinoid (Ro 13-6298) once per day for 4 days and found no changes in tibia trabecular BMD based on pQCT (Kneissel *et al.* 2005). Green *et al.* gavage-fed mice with ATRA (5 mg/kg/day) for 10 days and did not observe changes in trabecular bone volume of the tibia assessed by histomorphometric analysis (Green *et al.* 2015). On the other hand, Lind *et al.* observed a statistically significant decrease in femur trabecular BMD when rats were fed excess vitamin A (510 µg retinyl acetate and palmitate/g pellet) for 7 days (Lind *et al.* 2011), and Wray *et al.* also observed lower trabecular BMD of the tibia in rats fed adequate (4 µg retinyl palmitate/g chow) and supplemented (50 µg/g) vitamin A diets compared to marginal (0.35 µg/g) vitamin A diet from weaning to 2–3, 8–10 and 18–20 months of age (Wray *et al.* 2011). Likewise, C57BL/6 female mice fed a diet of 22.5 µg retinol/g of chow from weaning to 6 months of age had significantly reduced BV/TV in the vertebra, as demonstrated by Yorgan *et al.* (2016). This conflicting evidence of excess vitamin A on trabecular bone may partially be explained by administration, dosage, duration, gender and age differences of the rodents used in these investigations, but the effect of vitamin A on trabecular bone remains an area that has been relatively unexplored. We were unable to detect any effect on trabecular bone in the tibia or vertebra of female mice at 4 or 10 weeks using doses of vitamin A that decreased cortical bone mass, and our hypervitaminosis A dose did not significantly alter trabecular bone after 8 days.

A mechanism for the decrease in bone size was not elucidated by gene expression analysis on cortical bone or serum turnover markers. In agreement with these observations, the number of periosteal, endocortical and trabecular osteoclasts in the present study was not significantly affected with supplemented vitamin A diet. Kneissel *et al.* gave rats subcutaneous injections of 125 µg/kg of retinoid (Ro 13-6298) daily for 4 days. This caused an increase in serum TRAP5b levels due to increased periosteal osteoclast numbers (Kneissel *et al.* 2005). Lind *et al.* fed rats 510 µg retinyl acetate and palmitate/g pellet for 7 days and also found that a hypervitaminosis A dose increased the number of periosteal osteoclasts. In contrast to Kneissel *et al.*, Lind *et al.* observed that hypervitaminosis A decreased serum TRAP5b and CTX, possibly due to the reduced number of endocortical osteoclasts found in their study (Lind *et al.* 2011). Confirming the previous studies in rats (Kneissel *et al.* 2005, Lind *et al.* 2011), we found that short-term hypervitaminosis A in mice substantially increased osteoclast numbers on the periosteal surface of the long bones and increased serum Trap5b. The enhanced number of osteoclasts was associated with an increased expression of the genes encoding TRAP and cathepsin K due to the increased expression of the *RANKL* gene. This is in agreement with our previous *ex vivo* observations in vitamin A-treated mouse calvarial bones (Conaway *et al.* 2011). Similar to Lind *et al.*, we also observed that short term hypervitaminosis A, for reasons unknown, decreased endocortical osteoclast numbers.

Based on periosteal MS/BS in the femur and tibia of rats, studies have suggested that the number of active osteoblasts is decreased in hypervitaminosis A treatment (Kneissel *et al.* 2005, Lind *et al.* 2013). In one of these studies (Lind *et al.* 2013), a strong decrease of osteoblastic bone formation was seen in rats fed 510 µg retinyl acetate and palmitate/g pellet for 7 days. This resulted in a decrease in MAR and BFR in the periosteal bone of the femur. In another study (Kneissel *et al.* 2005), no decrease of MAR, but a decrease of MS was observed in rats given subcutaneous injections of Ro 13-6298. In the present study, we observed that the supplemental dose of vitamin A in mice also decreased periosteal bone formation at 8 days. In contrast to the observations in the periosteum, treatment with vitamin A for 8 days enhanced bone formation at the endocortical surface, with no change in MS/BS, suggesting increased osteoblast activity. Transient loss of this effect may indicate a new steady state in bone remodeling.

Altogether, our observations suggest that reduced periosteal circumference caused by vitamin A may be due to both increased bone resorption and decreased formation and that the reduced endocortical circumference may be due to both enhanced bone formation and reduced resorption. To elucidate the mechanism involved in the cortical bone phenotype in the present study, we used a bisphosphonate, ZA. Treating mice with ZA or a saline vehicle and supplemented or control vitamin A diets indicated that the effect of vitamin A on the amount of cortical bone seems to be primarily mediated by osteoclasts, because there was no effect on cortical bone parameters in mice fed vitamin A and treated with ZA. Our observations further suggest that the decreased periosteal bone formation and increased endocortical bone formation that were observed may be secondary to an effect of vitamin A on osteoclasts, but we cannot rule out the possibility that direct effects on bone formation are too small in magnitude to result in significant effects on cortical bone mass.

The present study was conducted in female mice, and since gender differences have been reported in age-related bone loss in mice (Glatt *et al.* 2007), our results may not necessarily be transferable to male mice. For the present study, we used young mice that were growing for the first 5–6 weeks of vitamin A dietary consumption. It is possible that the effects observed are a combination of bone growth and adult bone remodeling. However, we did not observe any vitamin A effects on bone length measurements. Although bone remodeling in growing mice may not be identical to bone remodeling in adult humans, our observations support the epidemiological findings in humans indicating that vitamin A is a risk factor for osteoporosis and further suggest that the UTL in humans may need to be re-evaluated in relation to the harmful effects on the skeleton.

## Conclusion

Our results suggest that even clinically relevant doses of vitamin A consumed over a long period of time may have a negative impact on bone phenotype, mainly on cortical bone, with trabecular bone remaining unaffected. These data might help to explain why increased vitamin A intake and enhanced serum retinol appear to be associated with an increased risk of non-vertebral fractures, mainly dependent on cortical bone, but not with risk of vertebral fractures, mainly dependent on trabecular bone.

## Supplementary Material

Supporting Figure 1

Supporting Figure 2

Supporting Table 1

Supporting Table 2

## Declaration of interest

The authors declare that there is no conflict of interest that could be perceived as prejudicing the impartiality of the research reported.

## Funding

This work was funded by the Marie Curie Initial Training Network (Euroclast, FP7-People-2013-ITN: #607446), the Swedish Research Council, the ALF/LUA research grant from the Sahlgrenska University Hospital in Gothenburg, the IngaBritt and Arne Lundberg Foundation, the Royal 80-Year Fund of King Gustav V and Åke Wibergs foundation.

## Author contribution statement

Author’s roles: Study design: U H L, H H C, P H and V L. Study conduct: V L, K L G, A W, S H W, and P H. Data collection: V L, A K, and J T. Data analysis: V L, H J, and P H. Data interpretation: V L, C O, P H, and U H L. Drafting manuscript: V L, P H, and U H L. Revising manuscript content: V L, C O, H H C, P H, and U H L. Approving final version of manuscript: V L, K L G, A W, S H W, A K, J T, H J, C O, H H C, P H, and U H L. U H L, P H and V L take responsibility for the integrity of the data analysis.
