# Supplementary material for: Clinically relevant doses of vitamin A decrease cortical bone mass in mice
Source: J Endocrinol. 2018 Sep 24;239(3):389–402. doi: 10.1530/JOE-18-0316 (PMC6215918; doi:10.1530/JOE-18-0316)
Supplement: Supporting Figure 1 [file joe-239-389-s001.pdf]

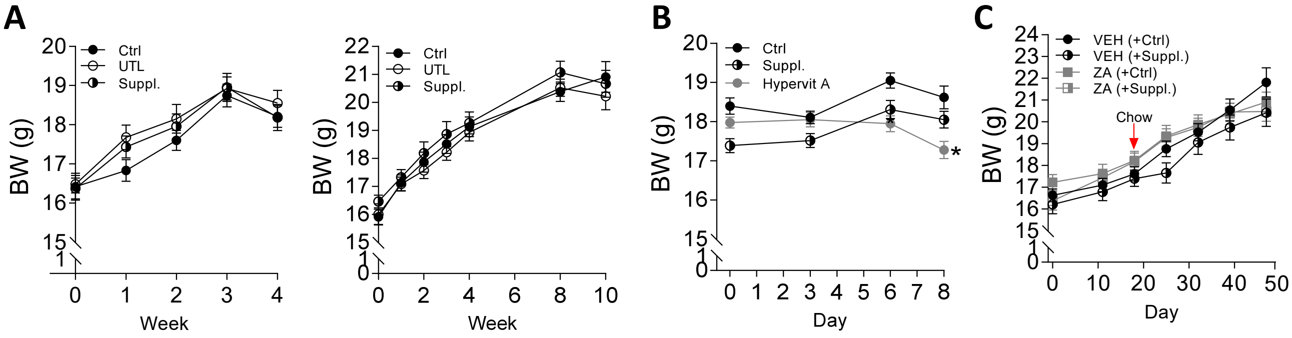

Supplementary Figure 1: Average body weight throughout the 4 week, 10 week, 8 day, and bisphosphonate experiments. Body weights (BW; g) monitored throughout the duration of the (A) 4- (n=15/group) and 10-week (n=10/group), (B) 8 day (n=10/group), and (C) bisphosphonate (n=10/group) experiments. Data in figures displayed as mean ± SEM . Differences in body weight increase from day 0, analysed by 1-way ANOVA with Dunnet's multiple comparison test, * $P < 0.05$ (A, B), or unpaired Student's t-test vs. respective control, $P > 0.05$ (C).
