# Supplementary material for: Clinically relevant doses of vitamin A decrease cortical bone mass in mice
Source: J Endocrinol. 2018 Sep 24;239(3):389–402. doi: 10.1530/JOE-18-0316 (PMC6215918; doi:10.1530/JOE-18-0316)
Supplement: Supporting Figure 2 [file joe-239-389-s002.pdf]

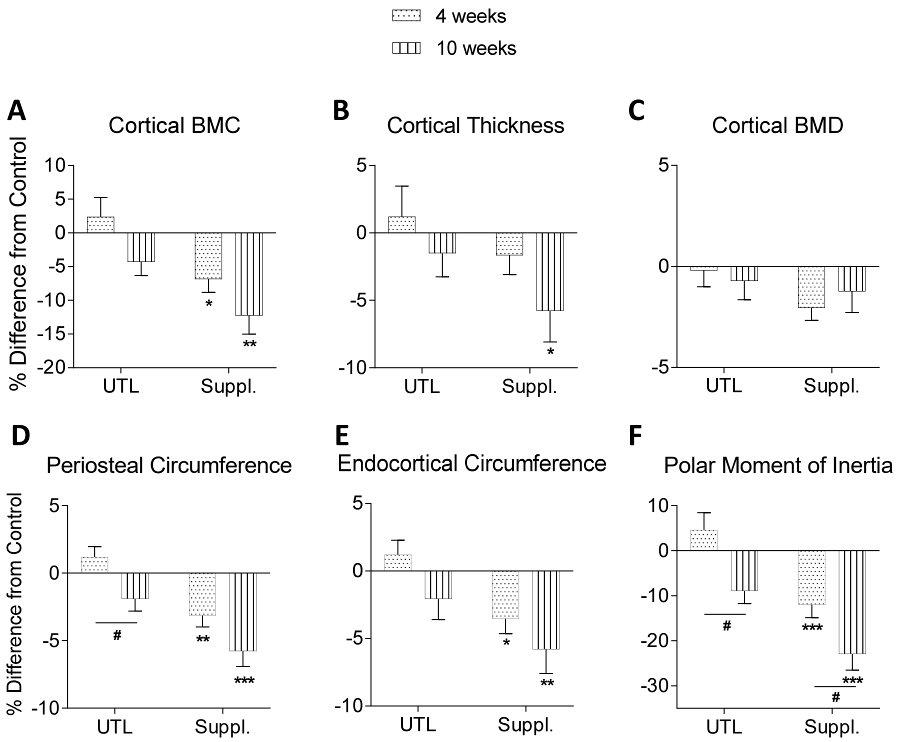

Supplementary Figure 2: Supplemented vitamin A and UTL dose affect cortical bone parameters of the femur in a time dependent manner. Femur pQCT results % difference vs. respective control after UTL and supplemented diets on (A) cortical bone mineral content (BMC), (B) cortical thickness, (C) cortical bone mineral density (BMD), (D) periosteal circumference, (E) endocortical circumference, and (F) polar moment of inertia. Values displayed as mean ± SEM, n=15/group at 4 weeks, n=10/group at 10 weeks. **P* < 0.05, ***P* < 0.01, ****P* < 0.001 Student's t-test vs. respective controls. #*P* < 0.05, 2-way ANOVA for interaction.
