# Supplementary material for: Clinically relevant doses of vitamin A decrease cortical bone mass in mice
Source: J Endocrinol. 2018 Sep 24;239(3):389–402. doi: 10.1530/JOE-18-0316 (PMC6215918; doi:10.1530/JOE-18-0316)
Supplement: Supporting Table 1 [file joe-239-389-t001.pdf]

Supplementary Table 1: Bone resorption and formation markers in serum indicate no effect of supplemented diet after 4 or 10 weeks.

	4 week			10 week		
	Control	Supplemental	CI of effect	Control	Supplemental	CI of effect
TRAP5b (U/L)	15.8 ± 1.0	18.8 ± 2.0	-2.05, 7.99	17.4 ± 2.7	17.8 ± 3.0	-7.91, 8.79
CTX (ng/ml)	25.5 ± 2.0	25.7 ± 2.0	-5.06, 6.06	22.0 ± 1.6	18.5 ± 1.7	-8.32, 1.36
Osteocalcin (ng/ml)	139.8 ± 11.2	130.9 ± 6.3	-34.5, 15.6	92.4 ± 4.7	75.5 ± 8.5	-37.4, 3.46

Serum TRAP5b, CTX, and osteocalcin levels after 4 and 10 weeks of supplemented vitamin A diet and the 95% confidence interval (CI) of the effect at each time point. Mean ± SEM, n=15/group at 4 weeks, n=10/group at 10 weeks, Student's t-test, $P > 0.05$.
