# Supplementary material for: Clinically relevant doses of vitamin A decrease cortical bone mass in mice
Source: J Endocrinol. 2018 Sep 24;239(3):389–402. doi: 10.1530/JOE-18-0316 (PMC6215918; doi:10.1530/JOE-18-0316)
Supplement: Supporting Table 2 [file joe-239-389-t002.pdf]

Supplementary Table 2: Histomorphometric analysis indicates no change in the number of osteoclasts present after 10 weeks of supplemented vitamin A diet.

	Peri. Oc.N/BS	Endo. Oc.N/BS	Trab. Oc.N/BS
Control (n=8)	0.22 ± 0.12	0.35 ± 0.11	0.28 ± 0.13
Supplemented (n=9)	0.17 ± 0.06	0.27 ± 0.06	0.42 ± 0.14
CI of effect	-0.33, 0.22	-0.34, 0.18	-0.27, 0.55

TRAP stained longitudinal sections of femur were taken and periosteal (Peri. Oc.N), endocortical (Endo. Oc.N) and trabecular (Trab. Oc.N) osteoclasts counted per measured bone surface (BS; mm^{-1}) and 95% confidence intervals (CI) of the effect. Data displayed as mean ± SEM, Student's t-test, $P > 0.05$.
